# A respiro-fermentative strategy to survive nanoxia in *Acidobacterium capsulatum*

**DOI:** 10.1093/femsec/fiae152

**Published:** 2024-11-18

**Authors:** Daniela Trojan, Emilio García-Robledo, Bela Hausmann, Niels Peter Revsbech, Dagmar Woebken, Stephanie A Eichorst

**Affiliations:** Department of Microbiology and Ecosystem Science, Centre for Microbiology and Environmental Systems Science, University of Vienna, 1030 Vienna, Austria; Department of Biology, Faculty of Marine and Environmental Sciences, University of Cádiz, 11002 Cádiz, Spain; Department of Microbiology and Ecosystem Science, Centre for Microbiology and Environmental Systems Science, University of Vienna, 1030 Vienna, Austria; Joint Microbiome Facility of the Medical University of Vienna and the University of Vienna, 1030 Vienna, Austria; Department of Laboratory Medicine, Medical University of Vienna, 1030 Vienna, Austria; WATEC, Department of Biology, Aarhus University, 8000 Aarhus, Denmark; Department of Microbiology and Ecosystem Science, Centre for Microbiology and Environmental Systems Science, University of Vienna, 1030 Vienna, Austria; Department of Microbiology and Ecosystem Science, Centre for Microbiology and Environmental Systems Science, University of Vienna, 1030 Vienna, Austria

**Keywords:** *Acidobacteriota*, oxygen limitation, microaerobic respiration, fermentation, NADH imbalances, transcriptome

## Abstract

Microbial soil habitats are characterized by rapid shifts in substrate and nutrient availabilities, as well as chemical and physical parameters. One such parameter that can vary in soil is oxygen; thus, microbial survival is dependent on adaptation to this substrate. To better understand the metabolic abilities and adaptive strategies to oxygen-deprived environments, we combined genomics with transcriptomics of a model organism, *Acidobacterium capsulatum*, to explore the effect of decreasing, environmentally relevant oxygen concentrations. The decrease from 10 to 0.1 µM oxygen (3.6 to 0.036 pO_2_% present atmospheric level, respectively) caused the upregulation of the transcription of genes involved in signal transduction mechanisms, energy production and conversion and secondary metabolites biosynthesis, transport, and catabolism based on clusters of orthologous group categories. Contrary to established observations for aerobic metabolism, key genes in oxidative stress response were significantly upregulated at lower oxygen concentrations, presumably due to an NADH/NAD^+^ redox ratio imbalance as the cells transitioned into nanoxia. Furthermore, *A. capsulatum* adapted to nanoxia by inducing a respiro-fermentative metabolism and rerouting fluxes of its central carbon and energy pathways to adapt to high NADH/NAD^+^ redox ratios. Our results reveal physiological features and metabolic capabilities that allowed *A. capsulatum* to adapt to oxygen-limited conditions, which could expand into other environmentally relevant soil strains.

## Teaser

Microorganisms face a multitude of fluctuating conditions in the environment (such as oxygen); *Acidobacteriumcapsulatum* has the flexibility to survive and thrive under these conditions.

## Introduction

Microorganisms face a multitude of fluctuating and often limiting conditions across various environments, such as soils, human gut, and aquatic environments. Carbon, electron acceptors (such as oxygen (O_2_)), and/or nutrients can vary over space and time. As such, microorganisms need to compensate and employ strategies to survive during these potentially growth-restricting conditions. One such strategy is respiratory flexibility. The utilization of both high- and low-affinity terminal oxidases enables exploitation of the full range of O_2_ concentrations for oxidative phosphorylation and energy conservation, providing a great benefit in the ever-changing O_2_ concentrations across environments. This can be attained by inducing branched respiratory chains that terminate in multiple oxidases with different affinities for O_2_ (Bueno et al. 2012) recently shown in members of ubiquitous soil bacteria, the *Acidobacteriota* (Eichorst et al. 2018, Trojan et al. 2021). Other strategies by which cells respond to limitations are, e.g. modifying enzyme synthesis to take up growth-limiting nutrients or by modulating uptake rates for nutrients available in excess (Roszak and Colwell 1987). Alternatively, they can reroute metabolic fluxes, which enables them to shift to alternative sources of energy and building blocks while avoiding possible blockages due to specific nutrient limitations (Roszak and Colwell 1987, Bergkessel et al. 2016).

Catabolism and ATP production are often incongruent during these periods of limitation (Stouthamer 1979). As a result of this incongruency, a trade-off can occur between catabolic rate and ATP yield, whereby bacteria utilize pathways for the most efficient production of molar ATP yield (Y_ATP_: mole of ATP/mole of oxidized substrate). For example, when catabolic rates are high but O_2_ limiting, fermentative pathways (when available) are employed, together with respiratory pathways, commonly referred to as respiro-fermentation physiology (Pfeiffer et al. 2001, Vemuri et al. 2007) allowing bacteria to maximize ATP production during electron acceptor limitation. This respiro-fermentative physiology has been observed in *Escherichia coli, Bacillus subtilis*, and *Saccharomyces cerevisiae*, yet the evolution and regulation of this metabolism is still under debate (Molenaar et al. 2009). Presumably, bacteria have evolved to harbor greater metabolic flexibility for ATP production, rather than pathways yielding optimal growth yield (Stouthamer 1979).

Members of the phylum *Acidobacteriota* are ubiquitous across numerous soils (Fierer 2017, Delgado-Baquerizo et al. 2018) with a central role in carbon mineralization and plant decomposition (Fierer 2017, Crowther et al. 2019). Still very little is known about factors controlling their abundance in the environment or their effects on biogeochemical cycles under changing environmental conditions. In this study, we investigated the adaptive capability to O_2_-limited conditions with a model member of the phylum *Acidobacteriota, Acidobacterium capsulatum* 161. It is a member of the family Acidobacteriaceae that is commonly found across many environments, such as soils. *Acidobacterium capsulatum* 161 has originally been documented to be capable of microaerophilic growth and only later of weak fermentative growth as well (Pankratov et al. 2012, Myers and King 2016). Recently, its capacity for respiratory flexibility due to the presence and functionality of high- and low-affinity terminal oxidases was demonstrated (Trojan et al. 2021).

Here, we expanded our investigation of this strain to ascertain if it has additional abilities to alter its metabolism, such as the rerouting of metabolic fluxes, by profiling the whole transcriptomic response of *A. capsulatum* 161 to decreasing O_2_ concentrations in the micro- and nanomolar range, the latter referred to as nanoxic (<1 µmol O_2_ l^−1^) (Berg et al. 2022). To date, no reports have closely documented the catabolic routes of carbon and energy metabolism of *Acidobacteriota* or have evaluated its global transcriptomic response to O_2_ deprivations. By combining genomics and hypoxic culture incubations using highly sensitive optical O_2_ sensors (Lehner et al. 2015), we were able to investigate transcription patterns at the oxic–anoxic interface and could observe a transition from respiratory to respiro-fermentative metabolism in *A. capsulatum* 161.

## Material and methods

### Growth conditions and experimental setup

As previously described (Trojan et al. 2021), *A. capsulatum* 161 (ATCC 51196, DSM 11244) was grown up in biological quadruplicates in a vitamins and salts base medium (Eichorst et al. 2007, 2011) amended with 10 mM glucose as the sole carbon source with a pH of 5 under fully aerated conditions at room temperature. The setup of the 225-min microoxic incubations using two LUMOS systems (Lehner et al. 2015) for O_2_ concentration monitoring and sample collection at discrete, declining O_2_ concentrations (10 µmol O_2_ l^−1^, 0.1 µmol O_2_ l^−1^, and 0.001 µmol O_2_ l^−1^) down to anoxia (0 µmol O_2_ l^−1^ is < 0.0005 µmol O_2_ l^−1^) was previously described in Trojan et al. (2021). Briefly, at select time points (Fig. S1), 30 ml of culture were collected for RNA extraction and transcriptome sequencing by a syringe. For an immediate inactivation, the syringes were prefilled with an acidic phenol-stop solution (Kits et al. 2014) and precooled at 4°C. After centrifugation, cell pellets were snap frozen in liquid nitrogen and then stored at –80°C.

### RNA extraction and purification

RNA was extracted from frozen cell pellets using an acidic phenol/chloroform/isoamyl alcohol protocol (Griffiths et al. 2000) with mechanical disruption (30 s, 4 ms^−1^, FastPrep-24 bead beater, MP Biomedicals, Heidelberg, Germany) (Trojan et al. 2021). Purification of RNA and verification of complete DNA removal were described previously (Trojan et al. 2021).

### Transcriptome sequencing

Triplicate RNA samples from selected O_2_ concentrations and time points were sent to the Vienna BioCenter Core Facilities for sequencing. rRNA was depleted using the NEB Ribo-Zero rRNA removal kit for bacteria. Sequencing was performed on an Illumina NextSeq 550 system resulting in a total of 8.2–18.2 million 75-nucleotide reads per sample; more details can be found in Trojan et al. (2021).

### Data processing and statistical analyses

Raw reads were trimmed of sequencing adapters and low-quality 3′ ends using BBduk (BBtools v37.61, https://jgi.doe.gov/data-and-tools/bbtools/) with default parameters and error-corrected using Bayes–Hammer module of SPAdes assembler version 3.13.0 (Nikolenko et al. 2013). Any reads mapping to either the SILVA SSU or LSU releases 132 (Quast et al. 2013) or the 5S rRNA database (Szymanski et al. 2016) with a sequence identity >70% (performed with BBmap, BBtools, https://jgi.doe.gov/data-and-tools/bbtools/) were removed from the dataset (Table S1). The remaining reads were mapped to the publicly available genome of the *Acidobacterium capsulatum* 161 (Eichorst et al. 2020). The RNA reads per gene were summarized using the featureCounts tool from the Subread package v1.6.2 (Liao et al. 2014). Based on the generated read count tables, transcripts per million (TPMs) were calculated in R v3.6.0. Differential expression analysis, such as the calculation of log_2_-fold changes of relative transcript abundance and the significance of these changes were calculated in DESeq2 v1.26.0 using default parameters and a *P*-value cutoff of .05 (Love et al. 2014).

## Results

### Global transcriptomic response under decreasing O_2_ concentrations

**Figure 1.**
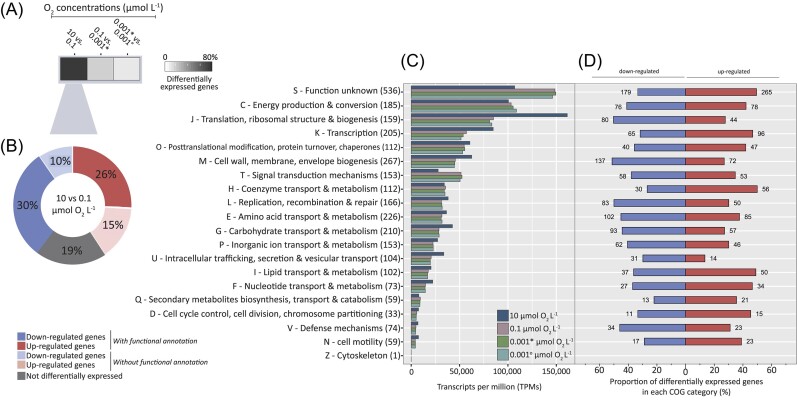
Impact of decreasing O_2_ concentrations on the transcriptome of *A. capsulatum* 161. (A) Heatmap depicts the proportion of genes that were differentially expressed (*P* < .05) between two O_2_ concentrations (µmol O_2_ l^−1^). The darker the color, the higher the proportion of genes whose expression has significantly changed between two O_2_ concentrations. (B) Breakdown of differentially transcribed genes (*P* ≤ .05) upon the decrease from 10 to 0.1 µmol O_2_ l^−1^. Downregulation is depicted in blue, upregulation in red. Proportion of genes encoding hypothetical proteins with no further functional annotation are shaded in light blue and light red, respectively. (C) The sum of TPM for all protein-coding genes transcribed in given COG categories in the transcriptomes at 10, 0.1, and 0.001 µmol O_2_ l^−1^. The number of transcribed genes per category is given in parentheses. Comparisons were done after 60 min at each respective O_2_ concentration with two exceptions: * depicts differential expression after 10 min, and ° depicts differential expression after 50 min. (D) Proportion of significantly differentially expressed genes upon the decrease from 10 to 0.1 µmol O_2_ l^−1^ for individual COG categories. Blue and red bars represent the percentages of genes showing lower and higher expression, respectively. Number of genes showing significantly differential higher or lower expression for each category is represented next to the bars. 0.001 µmol O_2_ l^−1^ represents an “apparent anoxia” as this concentration is close to the detection limit (0.0005 µmol O_2_ l^−1^) and could not be measured anymore, although 10.1 µmol O_2_ min^−1^ was still provided.

Our analyses revealed that the decrease from 10 to 0.1 µmol O_2_ l^−1^ had the greatest impact on gene expression with the highest number of significantly differentially expressed genes observed (Fig. 1A). Subsequent transitions to 0.001 and further to 0 µmol O_2_ l^−1^ invoked only a few to no significant expression changes (Fig. 1A). At 0.001 µmol O_2_ l^−1^, oxygen was still being supplied at 10.1 µmol O_2_ l^−1^ but could no longer be accurately determined, therefore is defined as “apparent anoxia.” Ninety-seven percent of all annotated genes (*n* = 3321 genes) were transcribed at least at one time point across the O_2_ concentrations. A significant difference in transcript numbers between 10 and 0.1 µmol O_2_ l^−1^ was detected for 2677 (∼81%) of transcribed genes. Of these genes, 41% were upregulated and 40% were downregulated at 0.1 µmol O_2_ l^−1^ (Fig. 1B). Genes encoding hypothetical proteins with no further functional annotation accounted for ∼25% of the differentially transcribed genes, whereas ∼26% and ∼30% of significantly upregulated and downregulated genes, respectively, were assigned to protein-coding genes with annotated function (Fig. 1B).

### Differential response of function-based categories to decreasing O_2_ concentrations

Genes that exhibited significant differential expression in response to O_2_ decrease were classified in clusters of orthologous groups (COGs) (Fig. 1C and D). The COG categories of energy production and conversion (C), amino acid transport and metabolism (E), nucleotide transport and metabolism (F), and lipid transport and metabolism (I) were the categories that were most altered by the decrease from 10 to 0.1 µmol O_2_ l^−1^ as more than 80% of the total genes assigned to these categories exhibited significant differential gene expression (Fig. 1D). More specifically, the transition from 10 to 0.1 µmol O_2_ l^−1^ reduced the number of TPMs in COGs related to translation, ribosomal structure, and biogenesis (J); transcription (K); carbohydrate transport and metabolism (G); cell wall, membrane, and envelope biogenesis (M); and intracellular trafficking, secretion, and vesicular transport (U) (Fig. 1C), and many of these categories had a higher proportion of significantly downregulated genes due to reduced O_2_ (Fig. 1D). Interestingly, the number of transcripts per million (TPMs) in COG categories pertaining to energy production and conversion (C), secondary metabolites biosynthesis, transport, and catabolism (Q), and signal transduction mechanisms (T) increased with decreasing O_2_ concentrations (Fig. 1C), and some of these categories also had a higher proportion of significantly upregulated genes due to reduced O_2_ (Fig. 1D).

### Universal and reactive oxygen stress response to decreasing O_2_

**Figure 2.**
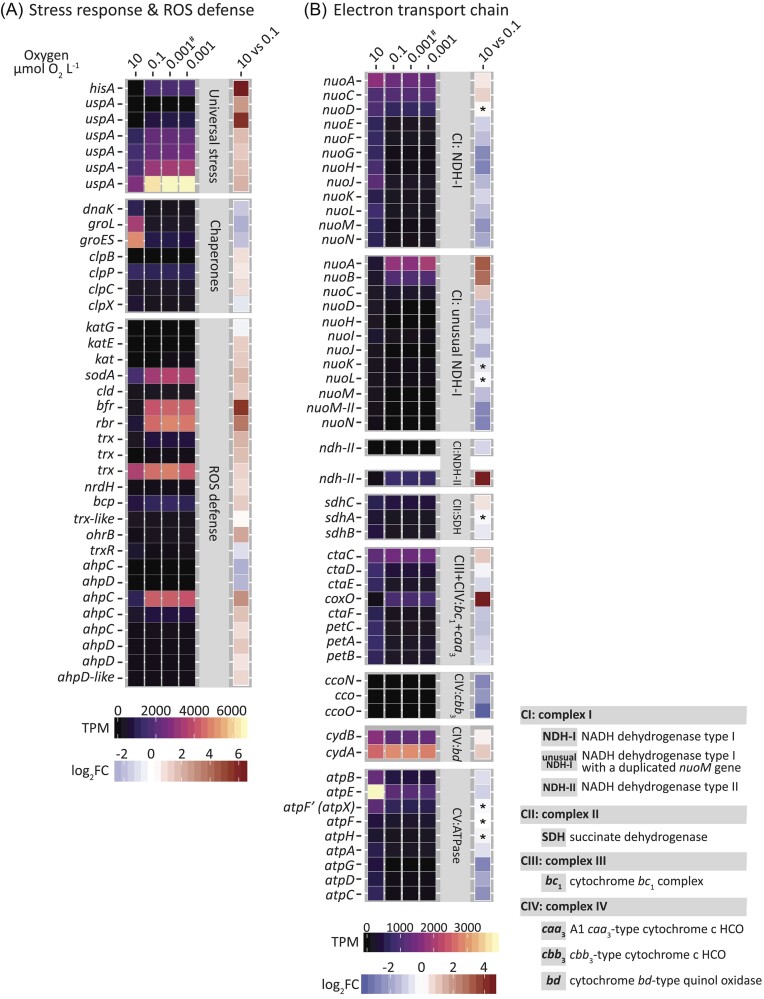
Transcription patterns of proteins involved in (A) stress response and ROS defense and (B) enzyme complexes in the ETC of *A. capsulatum* 161 exposed to decreasing O_2_ concentrations. Time-resolved gene expression at 10, 0.1, and 0.001 µmol O_2_ l^−1^ after 60 min at each respective O_2_ concentration with one exception: # depicts expression after 10 min at 0.001 µmol O_2_ l^−1^. Heatmaps depict average TPM values of biological replicates (*n* = 3). The last column depicts statistically significant differential (*P* ≤ .05) log_2_-fold changes (log_2_FC) of transcripts between 10 and 0.1 µmol O_2_ l^−1^ after 60 min at the respective O_2_ concentration. Downregulation is depicted in blue, upregulation in red. Asterisks depict non-significant differential expression (*P* > .05). 0.001 µmol O_2_ l^−1^ is defined as apparent anoxia: O_2_ was still supplied (10.1 µmol O_2_ min^−1^) but could no longer be accurately determined. Data for all replicates, gene locus tags, and further details are listed in Tables S2 and S3.

The transcription of genes encoding proteins involved in general stress response (COG category: T-signal transduction mechanisms) was significantly stimulated upon the decrease of O_2_ (Fig. 1C and D). In particular, a two-component sensor histidine kinase exhibited an 84-fold increase upon the shift to 0.1 µmol O_2_ l^−1^ (*P* ≤ .001) (Fig. 2A, Table S2). Furthermore, several homologs of the universal stress response gene *uspA* were significantly upregulated upon the decrease in oxygenation from 10 to 0.1 µmol O_2_ l^−1^. Four of those *uspA* genes had already been highly expressed at 10 µmol O_2_ l^−1^, whereas the expression of two of them, those with the highest fold increase (7- and 56-fold, respectively) seemed to be specifically stimulated by the drop to 0.1 µmol O_2_ l^−1^ (Fig. 2A, Table S2). The transcription levels of genes coding for various chaperone proteins differed between 10 and 0.1 µmol O_2_ l^−1^, but with no discernible trend of regulation (Fig. 2A, Table S2).

The transcriptome data at 10, 0.1, and 0.001 µmol O_2_ l^−1^ of the continuously decreasing O_2_ incubations exhibited the downregulation of key genes (*ahpCD, trxR, katG*) involved in oxidative stress defense (Fig. 2A, Table S2). Surprisingly, several other key genes coding for oxidative stress defense enzymes, catalase C (*katE*), non-heme manganese-containing catalase (*kat*), manganese superoxide dismutase (*soda*), heme-dependent chlorite dismutase (*cld*), rubrerythrin (*rbr*), thioredoxin (*trx*), ferroxidase (*bfr*), organic hydroperoxide resistance protein *ohrB*, and various homologs of alkyl hydroperoxide reductase subunits C and D (*ahpCD*) did not follow this trend and were transcribed at significantly higher levels at diminishing O_2_ concentrations (Fig. 2A, Table S2). Most of all, *rbr, oda, trx, bfr*, and one *ahpC* homolog (*ahpC-*2) were transcribed at high levels with an up to 13-fold upregulation upon the shift from 10 to 0.1 µmol O_2_ l^−1^ (Table S2).

### Expression of electron transport chain and oxidative phosphorylation

We detected gene expression for all complexes I–IV of the electron transport chain (ETC) and the ATP synthase (complex V) (Table S3). *Acidobacterium capsulatum* 161 harbors several complexes IV of the respiratory chain, and the transcriptional responses to the decrease in O_2_ from 10 to 0.1 µmol O_2_ l^−1^ (Fig. 2B, Table S3) were published and discussed recently (Trojan et al. 2021). The genes encoding the ATP synthase (complex V) were expressed during all O_2_-limiting conditions but showed a continuous decrease in transcription level with diminishing O_2_ availability (Fig. 2B, Table S3).

*Acidobacterium capsulatum* 161 expressed two proton-translocating NADH dehydrogenases (NDH-I, complex I). Out of the whole NDH-I *nuoA-N* operon, the *nuoAC* genes were continuously expressed at high transcription levels, whereas the transcription levels of other subunits (*nuoE-N*) decreased significantly with O_2_ (*P* ≤ .001; Fig. 2B, Table S3). The other NADH dehydrogenase was an unusual complex I (Chadwick et al. 2018), as it had a duplicated *nuo-M* gene. It had similar expression patterns as the NDH-I; the *nuoABC* transcripts were detected in significantly higher numbers at diminishing O_2_ concentrations (Fig. 2B, Table S3). Two homologs of the type II NADH dehydrogenase (NDH-II), which do not translocate protons across the cell membrane (Blaza et al. 2017), were transcribed. One homolog was detected at a very low level with a maximum TPM value of 10 at 10 µmol O_2_ l^−1^, whereas the second homolog was significantly higher expressed (*P* ≤ .001) upon the shift from 10 to 0.1 µmol O_2_ l^−1^ (21-fold), and this high expression was maintained at all subsequent O_2_ conditions with an average TPM value of 740 (Fig. 2B, Table S3). The operon (*sdhABC*) encoding the succinate dehydrogenase (SDH), complex II of the ETC, did not show any discernible pattern of regulation; e.g. the membrane subunit was upregulated, whereas the catalytic subunit (*sdhB*)was downregulated at 0.1 µmol O_2_ l^−1^ (Fig. 2B, Table S3).

### Decreasing O_2_ concentrations significantly altered the central metabolism

**Figure 3.**
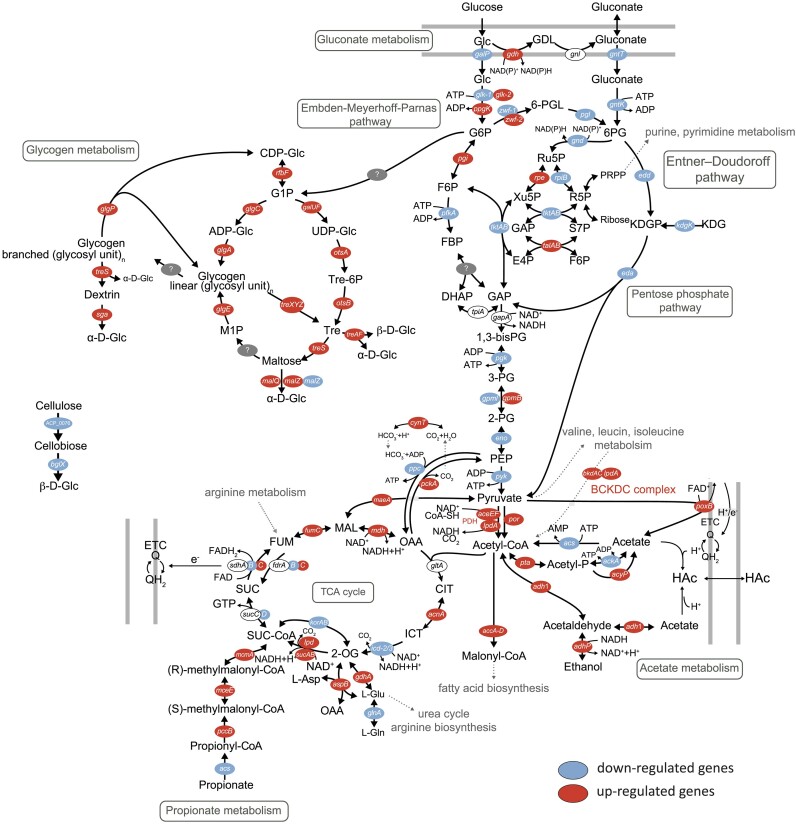
Differential gene expression due to low-nanomolar O_2_ concentrations across the central carbon and energy pathways of *A. capsulatum* 161 as inferred from analysis of the annotated genome and transcriptomes. Differential expression of genes involved in the EMP pathway, the ED pathway, the PP pathway, the TCA cycle as well as the gluconate-, acetate-, and glycogen metabolism. The genes represented in red were upregulated, while those represented in blue were downregulated upon the decrease from 10 to 0.1 µmol O_2_ l^−1^. Genes in white were non-significantly upregulated nor downregulated (*P* > .05). “?” depicts missing gene in the annotated genome. Expression of every gene was observed in all replicates (*n* = 3). The metabolite abbreviations are as follows: Glc, glucose; GDL, glucono-1,5-lactone; G6P, glucose-6-phosphate; 6-PGL, 6-phosphogluconolactone; 6PG, 6-phosphogluconate; KDPG, 2-keto-3-deoxy-6-phosphogluconate; KDG, 2-keto-3-deoxygluconate; Ru5P, ribulose 5-phosphate; R5P, ribose 5-phosphate; PRPP, phosphoribosyl diphosphate; Xu5P, xylulose 5-phosphate; S7P, sedoheptulose 7-phosphate; E4P, erythrose 4-phosphate; F6P, fructose 6-phosphate; FBP, fructose 1,6-bisphosphate; DHAP, dihydroxyacetone-3-phosphate; GAP, glyceraldehyde 3-phosphate; 1,3-bisPG, 1,3-biphosphoglycerate; 3-PG, 3-phosphoglycerate; 2-PG, 2-phosphoglycerate; PEP, phosphoenolpyruvate; Acetyl-P, acetyl phosphate; HAc, protonated form of acetate; CIT, citrate; ICT, isocitrate; 2-OG, 2-oxalglutarate, SUC-CoA, succinyl-coenzyme A; SUC, succinate; FUM, fumarate; MAL, malate; OAA, oxaloacetate; L-Asp, L-aspartate; L-Glu, L-glutamate; L-Gln, L-glutamine; CDP-Glc, cytidine diphosphate glucose; G1P, glucose-1-phosphate; UDP-Glc, uridine-5-diphosphate glucose; ADP-Glc, adenosine diphosphate glucose; Tre-6P, trehalose-6-phosphate; Tre, trehalose; M1P, maltose-1-phosphate; Q, quinone; QH_2_, quinol. ETC, electron transport chain. Data and abbreviations for all differentially expressed genes, gene locus tags, and further details are listed in Tables S2–S4.

With glucose in excess, decreasing O_2_ concentrations resulted in significant changes in the gene expression between 10 and 0.1 µmol O_2_ l^−1^ for the Embden–Meyerhof–Parnas (EMP) pathway, the Entner–Doudoroff (ED) pathway, the pentose phosphate (PP) pathway, genes of the pyruvate metabolism and the tricarboxylic acid (TCA) cycle as well as the acetate, glycogen, and gluconate metabolism (Fig. 3, Fig. S2, Table S4). More details are discussed below.

### Glucose transport and pyruvate production were downregulated at low-nanomolar O_2_ concentrations

**Figure 4.**
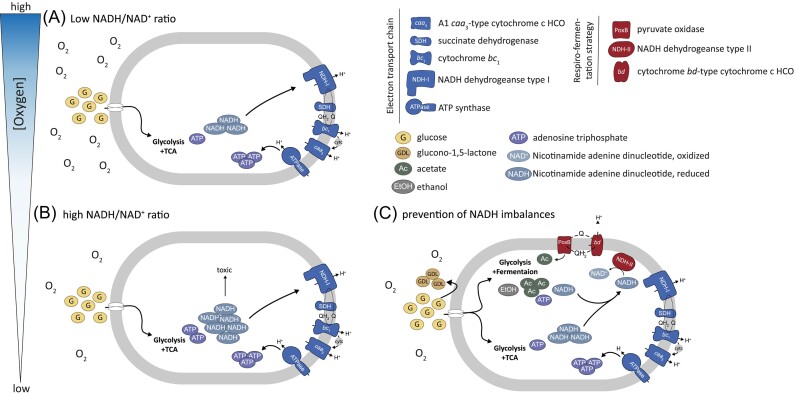
Conceptual figure depicting the response of *A. capsulatum* 161 to NADH/NAD^+^ ratio when transitioning from high to low nanomolar O_2_ concentrations and the associated physiological response. (A) At high O_2_ concentrations in glucose-unlimited medium, the ETC sufficiently consumes the NADH and electrons generated by glycolysis and TCA to keep the NADH/NAD^+^ redox ratio low. (B) Under O_2_-limited conditions, the ETC is limited by the lack of the terminal electron acceptor and cannot metabolize all the accumulated NADH, leading to NADH imbalances, ROS stress, and a potential toxicity. (C) By the onset of mixed acid fermentation, the production of acetate, and ethanol as well as shifting to the use of NDH-II rather than NDH-I, *A. capsulatum* 161 counteracts NADH imbalances and produces fewer NADH per ATP. It also oxidizes extracellular glucose to gluconate (glucono-1,5-lactone). Key for the abbreviations is depicted on the top right, except for the quinol/quinone cycle (QH_2_/Q) and cytochrome c (cytc). Figure adapted from Szenk et al. (2017).

The overall expression of genes in the glycolytic pathways decreased from 10 to 0.1 µmol O_2_ l^−1^ (Fig. 3, Fig. S2, Table S4). The transcription level of the symporter gene responsible for transporting glucose from the periplasm to the cytoplasm, *galP*, was significantly decreased (3.5-fold, *P* ≤ .001) upon the shift of oxygenation from 10 to 0.1 µmol O_2_ l^−1^ (Fig. 4, Fig. S1, Table S4).

The production of pyruvate was downregulated across various paths. Pyruvate stemming from 2-keto-3-deoxy-6-phosphogluconate (KDPG) in the ED pathway via KDPG aldolase (*eda* gene) (Bennett et al. 2009, Flamholz et al. 2013) was expressed at significantly lower levels (Fig. 3, Fig. S2, Table S4). And the *pfkA* gene, which is unique to the EMP pathway in the catabolic direction, was significantly downregulated at 0.1 µmol O_2_ l^−1^ (1.3-fold, Fig. S1, Table S4). The conversion of PEP to pyruvate, which is coupled to the synthesis of ATP, is catalyzed by pyruvate kinase (*pyk* gene) that exhibited a lower expression at diminishing O_2_ concentrations (Fig. S2, Table S4).

Apart from three genes, all genes involved in the PP pathway were significantly downregulated at 0.1 µmol O_2_ l^−1^ (Fig. 3, Fig. S2, Table S4).

### Pyruvate metabolism was highly transcribed at low-nanomolar O_2_ concentrations

Pyruvate occupies a key position in central carbon metabolism and is an important branch point between catabolic and biosynthetic pathways (Fig. 3). Genes encoding proteins involved in the pyruvate metabolism (such as *poxB, aceEF, lpdA, por*, and *maeA*) were transcribed at higher levels at lower O_2_ concentrations (Fig. 3, Fig. S2, Table S4).

### Upregulation of glycogen and downregulation of gluconate metabolism at lower O_2_ concentrations

Extracellular glucose can be oxidized to gluconate in the periplasm instead to G6P (Fig. 3). The membrane-bound glucose 1-dehydrogenase (*gdh*), which oxidizes glucose to glucono-1,5-lactone (Fig. 3), was one of the top upregulated genes (45-fold; log_2_-fold change of 5.5) and was transcribed at significantly higher levels at 0.1 µmol O_2_ l^−1^ (Fig. S1, Table S4). Yet gluconolaconase (*gnl*) had very low transcript levels at low O_2_ concentrations (Fig. 3, Table S4).

Another alternative route to the glycolytic pathways is glycogen metabolism. The genes that metabolize G6P to glycogen were upregulated in *A. capsulatum* 161. These glucose polymers can be stored and mobilized upon future demand. We found genes for both, biosynthesis as well as degradation of glycogen, in the genome of *A. capsulatum* 161 and genes encoding proteins involved in the glycogen metabolism were all upregulated upon the shift of O_2_ from 10 to 0.1 µmol O_2_ l^−1^ (Fig. 3, Fig. S2, Table S4).

### Differential transcriptional response of TCA cycle genes to O_2_ concentrations

Genes of the TCA cycle responded differently to the decrease of O_2_ (Fig. 3, Fig. S2, Table S4). The oxidative decarboxylation of acetyl-CoA is one of the main catalytic functions of the TCA cycle and provides reducing equivalents to the respiratory complexes. The first of four oxidative steps is a key rate-limiting step of the TCA cycle and is catalyzed by *icd*. The multiple homologs of the genes *icd* exhibited a high transcription level (high TPM levels) but were significantly downregulated at low-nanomolar O_2_ concentrations (Fig. S2, Table S4). In addition, the TCA cycle flux can be constricted by the availability of the oxaloacetate (OAA). The enzyme, phosphoenolpyruvate carboxykinase (*pckA*), was transcribed at significantly higher levels (7.5-fold) at 0.1 µmol O_2_ l^−1^, making OAA less available for the TCA cycle (Fig. 3).

However, the TCA cycle also functions in a biosynthetic capacity, primarily in the synthesis of amino acids, heme, and glucose. Glutamate and aspartate are synthesized from 2-oxoglutarate and OAA, respectively, via transamination, and both genes *gdhA* as well as *aspB*, exhibited an increased expression at 0.1 µmol O_2_ l^−1^. We detected neither the genes *aceA* and *aceB*, encoding the enzymes isocitrate lyase and malate synthase, nor the gene *glcB*, encoding the malate synthase G, neither in the genome nor in the transcriptome of *A. capsulatum* 161, which are involved in the glyoxylate shunt of the TCA cycle.

In addition to the oxidative conversion of pyruvate into acetyl-CoA, acetyl-CoA can also be synthesized through the catabolism of branched-chain amino acids by the branched-chain alpha-ketoacid dehydrogenase (BCKDC) complex (Fig. 3). The decrease in oxygenation to 0.1 µmol O_2_ l^−1^ increased the expression of this BCKDC complex up to 3.2-fold (Fig. 3, Fig. S2, Table S4).

### Significant transcriptional response of genes involved in production of acetate and ethanol due to decreasing O_2_ concentrations

Genes involved in various pathways for the production of acetate exhibited significantly higher expression levels at lower nanomolar O_2_ concentrations, such as phosphotransacetylase (*pta*) (Fig. 3, Fig. S2, Table S4). The conversion of acetate to acetyl-CoA via acetyl-CoA synthetase (*acs*) was downregulated at lower O_2_ concentrations (Fig. 3, Fig. S2, Table S4). The peripheral membrane protein pyruvate oxidase PoxB (also sometimes referred to as pyruvate dehydrogenase [ubiquinone]), which oxidatively decarboxylates pyruvate to form acetate and directly coupled to the respiratory chain (Gennis and Hager 1976, Koland et al. 1984, Abdel-Hamid et al. 2001) (Fig. 3), was one of the topmost upregulated genes of the central carbon and energy metabolism with a 45-fold increase in transcription at 0.1 µmol O_2_ l^−1^ (Fig. 3, Fig. S2, Table S4).

Apart from acetate production, ethanol production was significantly altered by decreasing O_2_ concentrations, and the homologs *adhP*, encoding for alcohol dehydrogenases, exhibited higher expression levels, up to 14.9-fold higher, at 0.1 µmol O_2_ l^−1^ (Fig. 3, Fig. S2, Table S4).

## Discussion

Adaptations and fast responses to changes in environmental conditions often occur at the metabolic level and in this work, we gained new insights into the transcription response of *A. capsulatum* 161 to diminishing O_2_ concentrations at low micro- and nanomolar levels. Our data indicate that diminishing O_2_ played a pivotal role in regulating the expression of genes involved in central metabolism under C excess conditions (Figs 3 and 4). To counter the toxic accumulation of respiration byproducts building up from the lack of O_2_, it shifted its metabolism and rerouted fluxes from an energy favorable respiratory state (Fig. 4B) to a respiro-fermentative condition, in which acetate together with ethanol seemed to be major end-products (Figs 3 and 4C).

Glucose transport, PP pathways, and pyruvate production were downregulated at low-nanomolar O_2_ concentrations (Fig. 3)—presumably to reduce the NADH/NAD^+^ redox ratio, which is a critical regulator of cell metabolism ultimately controlling the onset of respiro-fermentative metabolism (Shen and Atkinson 1970, Szenk et al. 2017). As *A. capsulatum* 161 transitioned from oxic to nanoxic conditions under C excess, the transcripts of many NADH-generating enzymes related to oxidative respiration were reduced (Fig. 4B). Glucose import (*galP)* exhibited reduced expression from 10 to 0.1 µmol O_2_ l^−1^, presumably as a means to limit the amount of available glucose. Yet, glucose 1-dehydrogenase *(gdh*) was overexpressed, potentially modulated a great part of the carbon flow through the gluconate bypass, thus reducing glucose concentration in the cell (Fig. 3). However, it appears that the cell did not use gluconate, as gluconolaconase had very low transcript levels at low O_2_ concentrations (<10 µmol O_2_ l^−1^). This could suggest that the enzyme requires a certain oxygen concentration to function. Pyruvate oxidase (PoxB) was upregulated in *A. capsulatum* 161, which catalyzes the decarboxylation of pyruvate to acetate and CO_2_ (Figs 3 and 4C), suggesting that pyruvate catabolism is the major switch point between the respiratory and fermentative responses.

The glycolytic flux was redirected toward the production of fermentation products, acetate (upregulation of *pta* and *acyP*) and ethanol (upregulation of *adhP*) (Figs 3 and 4C, Fig. S2, Table S4), to prevent carbon intermediates to enter the TCA cycle (El-Mansi and Holms 1989). Cells can then convert acetyl-CoA through the Pta-AckA pathway, producing and excreting acetate while generating ATP (El-Mansi and Holms 1989). Since the flux from acetyl-CoA to acetate does not generate any NADH (while the flux from acetyl-CoA through the TCA cycle generates 8 NAD(P)H and 2 FADH_2_), carbon flow diversion to acetate could be viewed as a means of *A. capsulatum* 161 to reduce or prevent further NADH accumulation (El-Mansi and Holms 1989, Holms 2001). This is in congruence with previous work on *Staphylococcus aureus*, where acetate production was enhanced under low O_2_ and glucose excess conditions (Ferreira et al. 2013). In addition, the conversion of acetaldehyde to ethanol via *adhP* was upregulated, consuming NADH and hence a way to counteract the NADH/NAD^+^ imbalance (Figs 3 and 4C).

Taken together, we hypothesize that the concomitant rise in NADH levels from glucose excess and low O_2_ conditions drove the onset of fermentative metabolism (acetate and ethanol production) to avoid toxic levels of NADH in the cell. Acetate and ethanol production stemming from pyruvate bypasses any energy-conserving steps associated with NADH, allowing a fast oxidation of pyruvate and efficient shuttling of protons/electrons to the ETC. Various studies have shown that in concentrated glucose environments, *E. coli* and other organisms switch to and obtain some of their energy anaerobically by acetate fermentation, even when O_2_ is plentiful, if the rate of glucose consumption is greater than the capacity to reoxidize the reduced equivalents generated (Farmer and Jones 1976, Hollywood and Doelle 1976, Andersen and Meyenburg 1980, Meyer et al. 1984, Farmer and Liao 1997, Kayser et al. 2005, Vemuri et al. 2006, Vazquez et al. 2008, Molenaar et al. 2009, Nahku et al. 2010, Valgepea et al. 2011, 2013, Zhuang et al. 2011, Basan et al. 2015, Peebo et al. 2015, Schütze et al. 2020). Although a major role of NADH is to supply electrons to the ETC thereby fueling the production of ATP, the strategy of *A. capsulatum* 161 was to reduce the NADH production stemming from respiratory pathways to avoid NADH imbalance while generating ATP (Szenk et al. 2017). The use of alternative pathways for NAD^+^ regeneration concomitant was also reported in other facultative anaerobes such as *E. coli* (Vemuri et al. 2006, Farhana et al. 2010, Martínez-Gómez et al. 2012, Szenk et al. 2017) and members of the genera *Salmonella* and *Shigella* (Gray et al. 1966, Wolfe 2005). The metabolic flexibility would allow these bacteria to cope with varying concentrations of carbon and O_2_ in such environments like soils. This respiro-fermentative strategy might extend into the *Acidobacteriota*, as many genomes harbor this potential as evidence by the presence of acetate kinase and alcohol dehydrogenase (Eichorst et al. 2018).

Our experimental conditions also invoked a significant upregulation of the glycogen metabolism suggesting cells transform excess glucose to the storage compound glycogen (Fig. 3, Fig. S2, Table S4). The accumulation of glycogen provides a metabolic reserve for *A. capsulatum* 161 under potential carbon-limited conditions in the future, which could be an important strategy in environments such as soils allowing cells to cope with transient limiting conditions.

Stress response to O_2_ and reactive oxygen species (ROS) is crucial for the ability to exist in habitats that are characterized by fluctuating O_2_ concentrations. Whether microbes can occupy such a habitat or a microniche within partly depends upon whether they are able to withstand local concentrations of high or low O_2_. Several universal stress proteins in *A. capsulatum* 161 were significantly upregulated by decreasing O_2_ concentrations approaching anoxia; especially two of these *usp* genes were affected by the drop of O_2_ to 0.1 µmol O_2_ l^−1^ (Fig. 2A, Table S2). Furthermore, the drop of O_2_ invoked a significant increase of transcription of a sensor histidine kinase (Fig. 2A, Table S2), which presumably allowed *A. capsulatum* 161 to sense environmental stimuli and manage various environmental changes by coupling environmental cues to gene expression (Stock et al. 2000, Mascher et al. 2006, Kaczmarczyk et al. 2014). Cellular stress can further lead to protein denaturation (Hightower 1991), and proteolytic removal of non‐functional proteins is crucial for optimal metabolic activities (Porankiewicz et al. 1999). We detected an upregulation of the ATP-dependent Clp proteases in the transcriptomic response of *A. capsulatum* 161 to diminishing O_2_ concentrations (Fig. 2A, Table S2), suggesting that they are important in removing irreversibly damaged polypeptides that may interfere with metabolic pathways under O_2_-limited stress conditions.

In *A. capsulatum* 161, a clear differential upregulation of genes involved in counteracting oxidative stress was observed upon the decrease of oxygenation from 10 to 0.1 µmol O_2_ l^−1^ (Fig. 2A, Table S2), indicating that it is capable of adapting to different redox states. Oxidative stress defense genes such as manganese superoxide dismutase, thioredoxins, and glutaredoxins were highly expressed under stimulated at low O_2_ (Fig. 2A, Table S2), as seen previously in *Nitrosomonas europaea* (Sedlacek et al. 2020). The increased demand for proteins involved in ROS defense could be caused by NADH/NAD^+^ redox ratio imbalances, as NADH accumulates and becomes toxic. Under O_2_-limiting conditions, an increased level of NADH builds up, as it is less efficiently reoxidized to NAD^+^ as a result of reduced aerobic respiration.

The high-affinity *bd*-type oxidase (*cydAB)* and NADH dehydrogenase (*ndh-II*) were upregulated upon the drop of oxygenation to 0.1 µmol O_2_ l^−1^ (Fig. 2B, Table S3). We previously hypothesized that the upregulation of the *bd*-type oxidase could suggest a contribution to respiratory activity at trace O_2_ conditions or favor the more faster electron flux than *cbb*_3_-type oxidases to permit more rapid reducing potential from carbon surplus (Trojan et al. 2021). The uncoupled NADH dehydrogenase NDH-II was highly upregulated at 0.1 µmol O_2_ l^−1^ (Fig. 2B, Table S3), presumably to compensate for the slow regeneration of NAD^+^ due to the low O_2_ availability. NDH-II only catalyzes the oxidation of NADH and reduction of quinones without the ability to pump protons, which, based on our data, seemed to be beneficial under micro- and nanoxic conditions. Alternative electron‐transfer routes seem to allow *A. capsulatum* 161 adjusting its energy transduction efficiency to its needs and substrate availability. In *A. capsulatum* 161, electrons can flow from the NADH dehydrogenase and SDH (complex II of the ETC) to the quinone/quinol pool, from where the electrons may either bypass the cytochrome *bc*_1_ complex (complex III) and directly flow to the *bd*-type quinol terminal oxidase or flow via a cytochrome c either to the low-affinity *caa*_3_‐type or the high-affinity *cbb*_3_‐type cytochrome c terminal oxidase. Under aerobic- or carbon-limiting conditions, the proton-coupled NDH-I might be the main driver of NADH oxidation and maintain the proton motive force required for ATP synthesis (Fig. 4A). Under the reduced environment in our incubations, when the reduction state of the quinone/quinol pool increases, the pathway to the NDH-II and *bd*-type quinol oxidase seemed to increase, with NDH-II being then the dominant dehydrogenase to oxidize excess NADH and support redox balance (Figs 2B and 4C). This strategy has been observed for *E. coli*, which may use NDH-II to counteract increasing NADH/NAD^+^ triggered by faster metabolism due to increased glucose uptake in order to support fast growth (Vemuri et al. 2006, Liu et al. 2019).

## Conclusions

In this study, we examined the transcriptional response of *A. capsulatum* 161 to diminishing O_2_ oncentrations in the low nanomolar range. Overall, O_2_-limiting conditions invoked a significant stress response in *A. capsulatum* 161. Our data indicate that *A. capsulatum* 161 has the genomic potential for multiple routes for the early steps of glucose catabolism. Under O_2_-limited but glucose-unlimited conditions, *A. capsulatum* 161 reroutes fluxes through its central metabolism from glycolysis to fermentative end products to counteract NADH/NAD^+^ imbalances building up due to loss of respiratory capacities under electron acceptor-limiting conditions. Understanding these capacities advances the knowledge on the metabolic responses *A. capsulatum* 161 is capable of in order to successfully thrive and persist under fluctuating substrate availabilities in terrestrial environments.

The investigated oxygen range (10 to 0.1 µmol O_2_ l^−1^ or 3.6–0.036 pO_2_% present atmospheric level) is environmentally relevant (Sexstone et al. 1985), presumably seen in various soil niches. Coping with dynamic O_2_ tensions is therefore vital for aerobic bacteria dwelling in (temporarily) O_2_-deprived habitats. During “spring snowmelt” or in the rhizosphere, catabolism and ATP yields can be uncoupled due to O_2_ limitation and carbon availability. To survive reductive stress during O_2_ deprivation, soil bacteria depend on metabolic strategies to maintain a proton motive force and redox balance. But modifications in metabolic routes at trace O_2_ levels extend beyond soils; these findings have implications in other environments, such as oxygen minimum zones (OMZs) in the Earth’s oceans. OMZs are large water masses with low oxygen concentrations, thus favoring anaerobic metabolism (Kalvelage et al. 2015). Interestingly, aerobic metabolism was previously detected in regions of apparent anoxic conditions (“anoxic” OMZs) (Garcia-Robledo et al. 2017), along with the presence of terminal oxidases (Kalvelage et al. 2015, Tsementzi et al. 2016) and production of O_2_ at trace levels (Canfield and Kraft 2022). These regions could provide a niche where bacteria transition from a respiratory to a respiro-fermentative metabolism to maximize energy yield, prior to using less favorable electron acceptors, such as nitrate. Taken together, the transition from aerobic respiration to a respiro-fermentative metabolism could provide bacteria the flexibility to generate energy during periods of limiting O_2_ in fluctuating environments for maintenance and survival of their populations.

## Supplementary Material

fiae152_Supplemental_Files

## Data Availability

The genome of *A. capsulatum* 161 is publicly available in the NCBI database with the following assembly accession number GCA_000022565.1. The raw transcriptomic reads are publicly available under BioProject accession number PRJNA635786.

## References

[bib1] Abdel-Hamid AM, Attwood MM, Guest JR. Pyruvate oxidase contributes to the aerobic growth efficiency of *Escherichia coli*. Microbiology. 2001;147:1483–98. 11390679 10.1099/00221287-147-6-1483

[bib2] Andersen KB, von Meyenburg K. Are growth rates of *Escherichia coli* in batch cultures limited by respiration?. J Bacteriol. 1980;144:114–23. 6998942 10.1128/jb.144.1.114-123.1980 PMC294601

[bib3] Basan M, Hui S, Okano H et al. Overflow metabolism in *Escherichia coli* results from efficient proteome allocation. Nature. 2015;528:99–104. 26632588 10.1038/nature15765 PMC4843128

[bib4] Bennett BD, Kimball EH, Gao M et al. Absolute metabolite concentrations and implied enzyme active site occupancy in *Escherichia coli*. Nat Chem Biol. 2009;5:593–9. 19561621 10.1038/nchembio.186 PMC2754216

[bib5] Berg JS, Ahmerkamp S, Pjevac P et al. How low can they go? Aerobic respiration by microorganisms under apparent anoxia. FEMS Microbiol Rev. 2022;46:fuac006. 35094062 10.1093/femsre/fuac006 PMC9075580

[bib6] Bergkessel M, Basta DW, Newman DK. The physiology of growth arrest: uniting molecular and environmental microbiology. Nat Rev Micro. 2016;14:549–63. 10.1038/nrmicro.2016.107 PMC10069271 27510862

[bib7] Blaza JN, Bridges HR, Aragão D et al. The mechanism of catalysis by type-II NADH:quinone oxidoreductases. Sci Rep. 2017;7:40165. 28067272 10.1038/srep40165 PMC5220320

[bib8] Bueno E, Mesa S, Bedmar EJ et al. Bacterial adaptation of respiration from xxic to microoxic and anoxic conditions: redox control. Antioxid Redox Signal. 2012;16:819–52. 22098259 10.1089/ars.2011.4051 PMC3283443

[bib9] Canfield DE, Kraft B. The ‘oxygen’ in oxygen minimum zones. Environ Microbiol. 2022;24:5332–44. 36054074 10.1111/1462-2920.16192 PMC9828761

[bib10] Chadwick GL, Hemp J, Fischer WW et al. Convergent evolution of unusual complex I homologs with increased proton pumping capacity: energetic and ecological implications. ISME J. 2018;12:2668–80. 29991762 10.1038/s41396-018-0210-1 PMC6194058

[bib11] Crowther TW, van den Hoogen J, Wan J et al. The global soil community and its influence on biogeochemistry. Science. 2019;365:eaav0550. 31439761 10.1126/science.aav0550

[bib12] Delgado-Baquerizo M, Oliverio AM, Brewer TE et al. A global atlas of the dominant bacteria found in soil. Science. 2018;359:320–5. 29348236 10.1126/science.aap9516

[bib13] Eichorst SA, Breznak JA, Schmidt TM. Isolation and characterization of soil bacteria that define *Terriglobus* gen. nov., in the phylum *Acidobacteria*. Appl Environ Microb. 2007;73:2708–17. 10.1128/AEM.02140-06 PMC1855589 17293520

[bib14] Eichorst SA, Kuske CR, Schmidt TM. Influence of plant polymers on the distribution and cultivation of bacteria in the phylum *Acidobacteria*. Appl Environ Microb. 2011;77:586–96. 10.1128/AEM.01080-10 PMC3020536 21097594

[bib15] Eichorst SA, Trojan D, Huntemann M et al. One complete and seven draft genome sequences of subdivision 1 and 3 *Acidobacteria* isolated from soil. Microbiol Resour Announc. 2020;9:e01087–19. 10.1128/MRA.01087-19 PMC6992861 32001557

[bib16] Eichorst SA, Trojan D, Roux S et al. Genomic insights into the *Acidobacteria* reveal strategies for their success in terrestrial environments. Environ Microbiol. 2018;20:1041–63. 29327410 10.1111/1462-2920.14043 PMC5900883

[bib17] El-Mansi EMT, Holms WH. Control of carbon flux to acetate excretion during growth of *Escherichia coli* in batch and continuous cultures. J Gen Microbiol. 1989;135:2875–83. 2693588 10.1099/00221287-135-11-2875

[bib18] Farhana A, Guidry L, Srivastava A et al. Reductive stress in microbes: implications for understanding *Mycobacterium tuberculosis* disease and persistence. Adv Microb Physiol. 2010;57:43–117. 21078441 10.1016/B978-0-12-381045-8.00002-3

[bib19] Farmer IS, Jones CW. The energetics of *Escherichia coli* during aerobic growth in continuous culture. Eur J Biochem. 1976;67:115–22. 786616 10.1111/j.1432-1033.1976.tb10639.x

[bib20] Farmer WR, Liao JC. Reduction of aerobic acetate production by *Escherichia coli*. Appl Environ Microb. 1997;63:3205–10. 10.1128/aem.63.8.3205-3210.1997 PMC168618 9251207

[bib21] Ferreira MT, Manso AS, Gaspar P et al. Effect of oxygen on glucose metabolism: utilization of lactate in *Staphylococcus aureus* as revealed by *in vivo* NMR studies. PLoS One. 2013;8:e58277. 23472168 10.1371/journal.pone.0058277 PMC3589339

[bib22] Fierer N. Embracing the unknown: disentangling the complexities of the soil microbiome. Nat Rev Micro. 2017;15:579–90. 10.1038/nrmicro.2017.87 28824177

[bib23] Flamholz A, Noor E, Bar-Even A et al. Glycolytic strategy as a tradeoff between energy yield and protein cost. Proc Natl Acad Sci USA. 2013;110:10039–44. 23630264 10.1073/pnas.1215283110 PMC3683749

[bib24] Garcia-Robledo E, Padilla CC, Aldunate M et al. Cryptic oxygen cycling in anoxic marine zones. Proc Nat Acad Sci USA. 2017;114:8319–24. 28716941 10.1073/pnas.1619844114 PMC5547588

[bib25] Gennis RB, Hager LP. Pyruvate Oxidase. In: Martinosi A (ed), The Enzymes of Biological Membranes, vol. 2. New York: Plenum, 1976, 493–504.

[bib26] Gray CT, Wimpenny JW, Mossman MR. Regulation of metabolism in facultative bacteria II. Effects of aerobiosis, anaerbiosis and nutrition on the formation of Krebs cycle enzymes in *Escherchia coli*. Biochim Biophys Acta. 1966;117:33–41. 5330664 10.1016/0304-4165(66)90149-8

[bib27] Griffiths RI, Whiteley AS, O'Donnell AG et al. Rapid method for coextraction of DNA and RNA from natural environments for analysis of ribosomal DNA- and rRNA-based microbial community composition. Appl Environ Microb. 2000;66:5488–91. 10.1128/aem.66.12.5488-5491.2000 PMC92488 11097934

[bib28] Hightower LE. Heat shock, stress proteins, chaperones, and proteotoxicity. Cell. 1991;66:191–7. 1855252 10.1016/0092-8674(91)90611-2

[bib29] Hollywood N, Doelle HW. Effect of specific growth rate and glucose concentration on growth and glucose metabolism of *Escherichia coli* K-12. Microbios. 1976;17:23–33. 801033

[bib30] Holms H. Flux analysis: a basic tool of microbial physiology. Adv Microb Physiol. 2001;45:271–340. 11450111 10.1016/s0065-2911(01)45006-5

[bib31] Kaczmarczyk A, Hochstrasser R, Vorholt JA et al. Complex two-component signaling regulates the general stress response in Alphaproteobacteria. Proc Natl Acad Sci USA. 2014;111:E5196–204. 25404331 10.1073/pnas.1410095111 PMC4260549

[bib32] Kalvelage T, Lavik G, Jensen MM et al. Aerobic microbial respiration in oceanic oxygen minimum zones. PLoS One. 2015;10:e0133526. 26192623 10.1371/journal.pone.0133526 PMC4507870

[bib33] Kayser A, Weber J, Hecht V et al. Metabolic flux analysis of *Escherichia coli* in glucose-limited continuous culture. I. Growth-rate-dependent metabolic efficiency at steady state. Microbiology. 2005;151:693–706. 15758216 10.1099/mic.0.27481-0

[bib34] Kits KD, Klotz MG, Stein LY. Methane oxidation coupled to nitrate reduction under hypoxia by the gammaproteobacterium *Methylomonas denitrificans*, sp. nov. Type strain FJG1. Environ Microbiol. 2014;17:3219–32. 10.1111/1462-2920.12772 25580993

[bib35] Koland JG, Miller MJ, Gennis RB. Reconstitution of the membrane-bound, ubiquinone-dependent pyruvate oxidase respiratory chain of *Escherichia coli* with the cytochrome d terminal oxidase. Biochemistry. 1984;23:445–53. 6367818 10.1021/bi00298a008

[bib36] Lehner P, Larndorfer C, Garcia-Robledo E et al. LUMOS—a sensitive and reliable optode system for measuring dissolved oxygen in the nanomolar range. PLoS One. 2015;10:e0128125. 26029920 10.1371/journal.pone.0128125 PMC4451986

[bib37] Liao Y, Smyth GK, Shi W. featureCounts: an efficient general purpose program for assigning sequence reads to genomic features. Bioinformatics. 2014;30:923–30. 24227677 10.1093/bioinformatics/btt656

[bib38] Liu Y, Landick R, Raman S. A regulatory NADH/NAD+ redox biosensor for bacteria. ACS Synth Biol. 2019;8:264–73. 30633862 10.1021/acssynbio.8b00485

[bib39] Love MI, Huber W, Anders S. Moderated estimation of fold change and dispersion for RNA-seq data with DESeq2. Genome Biol. 2014;15:550–21. 25516281 10.1186/s13059-014-0550-8 PMC4302049

[bib40] Martínez-Gómez K, Flores N, Castañeda HM et al. New insights into *Escherichia coli* metabolism: carbon scavenging, acetate metabolism and carbon recycling responses during growth on glycerol. Microb Cell Fact. 2012;11:46. 22513097 10.1186/1475-2859-11-46 PMC3390287

[bib41] Mascher T, Helmann JD, Unden G. Stimulus perception in bacterial signal-transducing histidine kinases. Microbiol Mol Biol Rev. 2006;70:910–38. 17158704 10.1128/MMBR.00020-06 PMC1698512

[bib42] Meyer H-P, Leist C, Fiechter A. Acetate formation in continuous culture of *Escherichia coli* K12 D1 on defined and complex media. J Biotechnol. 1984;1:355–8.

[bib43] Molenaar D, van Berlo R, de Ridder D et al. Shifts in growth strategies reflect tradeoffs in cellular economics. Mol Syst Biol. 2009;5:323. 19888218 10.1038/msb.2009.82 PMC2795476

[bib44] Myers MR, King GM. Isolation and characterization of *Acidobacterium ailaaui* sp. nov., a novel member of *Acidobacteria* sub-division I, from a geothermally-heated Hawaiian microbial mat. Int J Syst Evol Microbiol. 2016;66:5328–35. 27692038 10.1099/ijsem.0.001516

[bib45] Nahku R, Valgepea K, Lahtvee P-J et al. Specific growth rate dependent transcriptome profiling of *Escherichia coli* K12 MG1655 in accelerostat cultures. J Biotechnol. 2010;145:60–5. 19861135 10.1016/j.jbiotec.2009.10.007

[bib46] Nikolenko SI, Korobeynikov AI, Alekseyev MA. BayesHammer: Bayesian clustering for error correction in single-cell sequencing. BMC Genomics. 2013;14 Suppl 1:S7–11. 10.1186/1471-2164-14-S1-S7 PMC3549815 23368723

[bib47] Pankratov TA, Kirsanova LA, Kaparullina EN et al. *Telmatobacter bradus* gen. nov., sp. nov., a cellulolytic facultative anaerobe from subdivision 1 of the *Acidobacteria* and emended description of *Acidobacterium capsulatum* Kishimoto et al. 1991. Int J Syst Evol Microbiol. 2012;62:430–7. 21460138 10.1099/ijs.0.029629-0

[bib48] Peebo K, Valgepea K, Maser A et al. Proteome reallocation in *Escherichia coli* with increasing specific growth rate. Mol Biosyst. 2015;11:1184–93. 25712329 10.1039/c4mb00721b

[bib49] Pfeiffer T, Schuster S, Bonhoeffer S. Cooperation and competition in the evolution of ATP-producing pathways. Science. 2001;292:504–7. 11283355 10.1126/science.1058079

[bib50] Porankiewicz J, Wang J, Clarke AK. New insights into the ATP-dependent clp protease: *E scherichia coli* and beyond. Mol Microbiol. 1999;32:449–58. 10320569 10.1046/j.1365-2958.1999.01357.x

[bib51] Quast C, Pruesse E, Yilmaz P et al. The SILVA ribosomal RNA gene database project: improved data processing and web-based tools. Nucleic Acids Res. 2013;41:D590–6. 23193283 10.1093/nar/gks1219 PMC3531112

[bib52] Roszak DB, Colwell RR. Survival strategies of bacteria in the natural environment. Microbiol Rev. 1987;51:365–79. 3312987 10.1128/mr.51.3.365-379.1987 PMC373117

[bib53] Schütze A, Benndorf D, Püttker S et al. The impact of *ackA, pta*, and *ackA-pta* mutations on growth, gene expression and protein acetylation in *Escherichia coli* K-12. Front Microbiol. 2020;11:233. 32153530 10.3389/fmicb.2020.00233 PMC7047895

[bib54] Sedlacek CJ, Giguere AT, Dobie MD et al. Transcriptomic response of *Nitrosomonas europaea* transitioned from ammonia- to oxygen-limited steady-state growth. Msystems. 2020;5:e00562–19. 31937676 10.1128/mSystems.00562-19 PMC6967387

[bib55] Sexstone AJ, Revsbach NP, Parkin TB et al. Direct measurement of oxygen profiles and denitrification rates in soil aggregates. Soil Microbiol Biochem. 1985;49:645–51.

[bib56] Shen LC, Atkinson DE. Regulation of pyruvate dehydrogenase from *Escherichia coli*. Interactions of adenylate energy charge and other regulatory parameters. J Biol Chem. 1970;245:5974–8. 4320794

[bib57] Stock AM, Robinson VL, Goudreau PN. Two-component signal transduction. Annu Rev Biochem. 2000;69:183–215. 10966457 10.1146/annurev.biochem.69.1.183

[bib58] Stouthamer AH. The search for correlation between theoretical and experimental growth yields. In: Quayle JR (ed.) International Review of Biochemistry. Microbial Biochemistryvol. 21. Baltimore: University Park Press, 1979. 1–46.

[bib59] Szenk M, Dill KA, de Graff AMR. Why do fast-growing bacteria enter overflow metabolism? Testing the membrane real estate hypothesis. Cell Syst. 2017;5:95–104. 28755958 10.1016/j.cels.2017.06.005

[bib60] Szymanski M, Zielezinski A, Barciszewski J et al. 5SRNAdb: an information resource for 5S ribosomal RNAs. Nucleic Acids Res. 2016;44:D180–3. 26490961 10.1093/nar/gkv1081 PMC4702797

[bib61] Trojan D, Garcia-Robledo E, Meier DV et al. Microaerobic lifestyle at nanomolar O_2_ concentrations mediated by low-affinity terminal oxidases in abundant soil bacteria. Msystems. 2021;6:e00250–21. 34227829 10.1128/mSystems.00250-21 PMC8407424

[bib62] Tsementzi D, Wu J, Deutsch S et al. SAR11 bacteria linked to ocean anoxia and nitrogen loss. Nature. 2016;536:179–83. 27487207 10.1038/nature19068 PMC4990128

[bib63] Valgepea K, Adamberg K, Seiman A et al. *Escherichia coli* achieves faster growth by increasing catalytic and translation rates of proteins. Mol Biosyst. 2013;9:2344–58. 23824091 10.1039/c3mb70119k

[bib64] Valgepea K, Adamberg K, Vilu R. Decrease of energy spilling in *Escherichia coli* continuous cultures with rising specific growth rate and carbon wasting. BMC Syst Biol. 2011;5:106. 21726468 10.1186/1752-0509-5-106 PMC3149000

[bib65] Vazquez A, Beg QK, deMenezes MA et al. Impact of the solvent capacity constraint on *E. coli* metabolism. BMC Syst Biol. 2008;2:7. 18215292 10.1186/1752-0509-2-7 PMC2270259

[bib66] Vemuri GN, Altman E, Sangurdekar DP et al. Overflow metabolism in *Escherichia coli* during steady-state growth: transcriptional regulation and effect of the redox ratio. Appl Environ Microb. 2006;72:3653–61. 10.1128/AEM.72.5.3653-3661.2006 PMC1472329 16672514

[bib67] Vemuri GN, Eiteman MA, McEwen JE et al. Increasing NADH oxidation reduces overflow metabolism in *Saccharomyces cerevisiae*. Proc Natl Acad Sci USA. 2007;104:2402–7. 17287356 10.1073/pnas.0607469104 PMC1892921

[bib68] Wolfe AJ. The acetate switch. Microbiol Mol Biol Rev. 2005;69:12–50. 15755952 10.1128/MMBR.69.1.12-50.2005 PMC1082793

[bib69] Zhuang K, Vemuri GN, Mahadevan R. Economics of membrane occupancy and respiro-fermentation. Mol Syst Biol. 2011;7:500. 21694717 10.1038/msb.2011.34 PMC3159977

