# Supplementary material for: A respiro-fermentative strategy to survive nanoxia in Acidobacterium capsulatum
Source: FEMS Microbiol Ecol. 2024 Nov 18;100(12):fiae152. doi: 10.1093/femsec/fiae152 (PMC11636273; doi:10.1093/femsec/fiae152)
Supplement: fiae152_Supplemental_Files [file fiae152_supplemental_files.zip › Trojan_FigureLegends_TrackChanges.docx]

**FIGURE LEGENDS**

**Figure 1. Impact of decreasing O_2_ concentrations on the transcriptome of *Acidobacterium capsulatum* 161. (A)** Heatmap depicts the proportion of genes that were differentially expressed (*P* < 0.05) between two O_2_ concentrations (µmol O_2_ L^-1^). The darker the color, the higher the proportion of genes which expression has significantly changed between two O_2_ concentrations. **(B)** Breakdown of differentially transcribed genes (*P* ≤ 0.05) upon the decrease from 10 to 0.1 µmol O_2_ L^-1^. Down-regulation is depicted in blue, up-regulation in red. Proportion of genes encoding hypothetical proteins with no further functional annotation are shaded in light blue and light red, respectively. **(C)** The sum of transcripts per million (TPM) for all protein-coding genes transcribed in given COG categories in the transcriptomes at 10, 0.1 and 0.001 µmol O_2_ L^-1^. The number of transcribed genes per category is given in parentheses. Comparisons were done after 60 minutes at each respective O_2_ concentration with two exceptions: *depicts differential expression after 10 minutes and °depicts differential expression after 50 minutes. **(D)** Proportion of significantly differentially expressed genes upon the decrease from 10 to 0.1 µmol O_2_ L^-1^ for individual COG categories. Blue and red bars represent the percentages of genes showing lower and higher expression, respectively. Number of genes showing significantly differential higher or lower expression for each category is represented next to the bars. 0.001 µmol O_2_ L^-1^ represents an “apparent anoxia” as this concentration is close to the detection limit (0.0005 µmol O_2_ L^-1^) and could not be measured anymore although 10.1 µmol O_2_ min^-1^ was still provided.

**Figure 2. Transcription patterns of proteins involved in (A) stress response and reactive oxygen species (ROS) defense and (B) enzyme complexes in the electron transport chain of *Acidobacterium capsulatum* 161 exposed to decreasing O_2_ concentrations.** Time-resolved gene expression at 10, 0.1 and 0.001 µmol O_2_ L^-1^ after 60 min at each respective O_2_ concentration with one exception: # depicts expression after 10 min at 0.001 µmol O_2_ L^-1^. Heatmaps depicts average transcript per million (TPM) values of biological replicates (n = 3). The last column depicts statistically significant differential (*P* ≤ 0.05) log_2_ fold changes (log_2_FC) of transcripts between 10 and 0.1 µmol O_2_ L^-1^ after 60 minutes at the respective O_2_ concentration. Down-regulation is depicted in blue, up-regulation in red. Asterisks depict non-significant differential expression (*P* > 0.05). 0.001 µmol O_2_ L^-1^ is defined as apparent anoxia: O_2_ was still supplied (10.1 µmol O_2_ min^-1^) but could no longer be accurately determined. Data for all replicates, gene locus tags and further details are listed in **Tables S2 & 3**.

**Figure 3. Differential gene expression due to low nanomolar O_2_ concentrations across the central carbon and energy pathways of *Acidobacterium capsulatum* 161 as inferred from analysis of the annotated genome and transcriptome.** Differential expression of genes involved in the Embden–Meyerhof–Parnas pathway, the Entner–Doudoroff pathway, the pentose phosphate pathway, the tricarboxylic acid cycle as well as the gluconate-, acetate-, and glycogen metabolism. The genes represented in red were upregulated while those represented in blue were downregulated upon the decrease from 10 to 0.1 µmol O_2_ L^-1^. Genes in white were non-significantly up- nor down-regulated (*P* > 0.05). “?” depicts missing gene in the annotated genome. Expression of every gene was observed in all replicates (n = 3). The metabolite abbreviations are as follows: Glc, glucose; GDL, glucono-1,5-lactone; G6P, glucose-6-phosphate; 6-PGL, 6-phosphogluconolactone; 6PG, 6-phosphogluconate; KDPG, 2-keto-3-deoxy-6-phosphogluconate; KDG, 2-keto-3-deoxygluconate; Ru5P, ribulose 5-phosphate; R5P, ribose 5-phosphate; PRPP, phosphoribosyl diphosphate; Xu5P, xylulose 5-phosphate; S7P, sedoheptulose 7-phosphate; E4P, erythrose 4-phosphate; F6P, fructose 6-phosphate; FBP, fructose 1,6-bisphosphate; DHAP, dihydroxyacetone-3-phosphate; GAP, glyceraldehyde 3-phosphate; 1,3-bisPG, 1,3-biphosphoglycerate; 3-PG, 3-phosphoglycerate; 2-PG, 2-phosphoglycerate; PEP, phosphoenolpyruvate; Acetyl-P, acetyl phosphate; HAc, protonated form of acetate; CIT, citrate; ICT, isocitrate; 2-OG, 2-oxalglutarate, SUC-CoA, succinyl-coenzyme A; SUC, succinate; FUM, fumarate; MAL, malate; OAA, oxaloacetate; L-Asp, L-aspartate; L-Glu, L-Glutamate; L-Gln, L-Glutamine; CDP-Glc, cytidine diphosphate glucose; G1P, glucose-1-phosphate; UDP-Glc, uridine-5-diphosphate glucose; ADP-Glc, adenosine diphosphate glucose; Tre-6P, trehalose-6-phosphate; Tre, trehalose; M1P, maltose-1-phosphate; Q, quinone; QH_2_, quinol. ETC, electron transport chain. Data and abbreviations for all differentially expressed genes, gene locus tags and further details are listed in **Table S2-4**.

**Figure 4. Conceptual figure depicting the response of *Acidobacterium capsulatum* 161 to NADH/NAD^+^ ratio when transitioning from high to low nanomolar O_2_ concentrations and the associated physiological response.** **(A)** At high O_2_ concentrations in glucose-unlimited media, the electron transport chain (ETC) sufficiently consumes the NADH and electrons generated by glycolysis and tricarboxylic acid cycle (TCA) to keep the NADH/NAD^+^ redox ratio low. **(B)** Under O_2_-limited conditions, the ETC is limited by the lack of the terminal electron acceptor and cannot metabolize all the accumulated NADH, leading to NADH imbalances, ROS stress and a potential toxicity. **(C)** By the onset of mixed acid fermentation, the production of acetate and ethanol as well as shifting to the use of NDH-II rather than NDH-I, *Acidobacterium capsulatum* 161 counteracts NADH imbalances and produces fewer NADH per ATP. It also oxidizes extracellular glucose to gluconate (glucono-1,5-lactone). Key for the abbreviations is depicted on the top, right except for the quinol/quinone cycle (QH_2_/Q) and cytochrome c (cytc). Figure adapted from (Szenk, Dill and Graff 2017).
