# Supplementary material for: A respiro-fermentative strategy to survive nanoxia in Acidobacterium capsulatum
Source: FEMS Microbiol Ecol. 2024 Nov 18;100(12):fiae152. doi: 10.1093/femsec/fiae152 (PMC11636273; doi:10.1093/femsec/fiae152)
Supplement: fiae152_Supplemental_Files [file fiae152_supplemental_files.zip › Trojan_Supplemental_Material.pdf]

SUPPLEMENTAL MATERIAL

A respiro-fermentative strategy to survive nanoxia in *Acidobacterium capsulatum*

Daniela Trojan, Emilio García-Robledo, Bela Hausmann, Niels Peter Revsbech, Dagmar Woebken, Stephanie A. Eichorst

Address correspondence to Stephanie A. Eichorst, stephanie.eichorst@univie.ac.at

This PDF file includes:

Figure S1
Figure S2
Table S1

Other supplement material for this manuscript include the following:

Datasets Table S2, S3 and S4, XLSX file

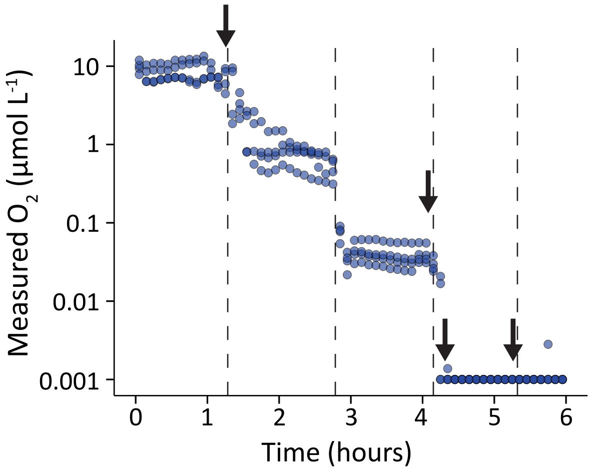

Figure S1. Sampling scheme. Measured O_2 concentrations in cultures of *Acidobacterium capsulatum* 161 over time (n = 4 biological replicates) during O_2-limited incubations. O_2 was decreased in a stepwise manner from 10 to 1 to 0.1 to 0.001 to 0 µmol O_2 L^{-1}. Vertical dashed lines depict the transition time points, while arrows indicate transcriptome sampling points after 60 or 15 minutes at the respective O_2 concentration. 0.001 µmol O_2 L^{-1} is defined as apparent anoxia: $O_2$was still supplied (10.1 µmol O_2 min^{-1}) but could no longer be accurately determined. A concentration of 0 µmol O_2 L^{-1} indicates no O_2 supply. The detection limit of Lumos was 0.0005 µmol O_2 L^{-1}; hence, 0 µmol O_2 $liter^{-1}$ is <0.0005 µmol O_2 L^{-1}. For further details, see Material and Methods.

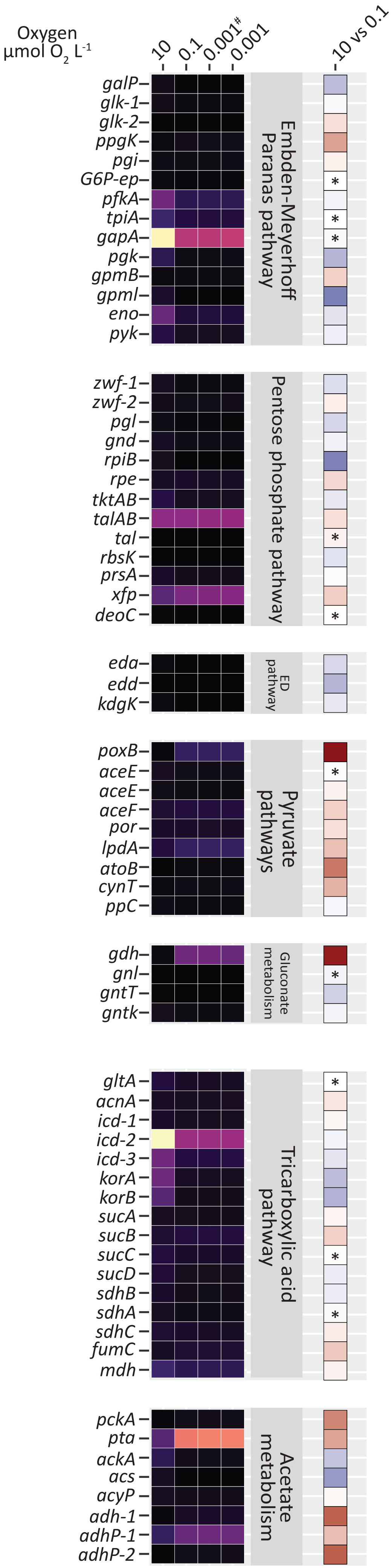

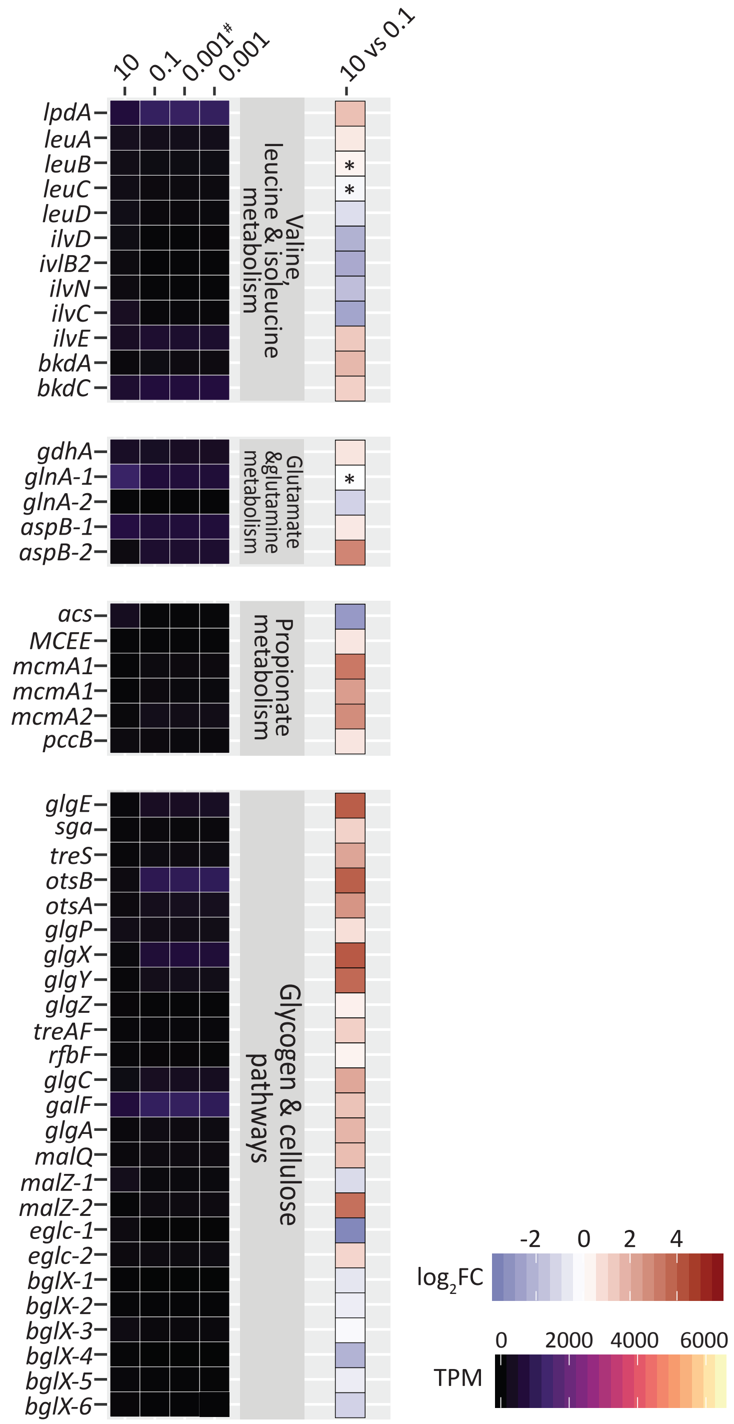

Figure S2. Transcription patterns of proteins of the central carbon and energy pathways of *Acidobacterium capsulatum* 161 exposed to decreasing O_2 concentrations. Time-resolved gene expression at 10, 0.1, 0.001 and 0 µmol O_2 L^{-1} after 60 min at each respective O_2 concentration with one exception: #depicts expression after 10 min at 0.001 µmol O_2 L^{-1}. Heatmaps show average transcript per million (TPM) values of biological replicates (n= 3). Locus tags and gene names are listed. The last column depicts statistically significant ($P \leq 0.05$) $\log_2$ fold changes ($\log_2$FC) of transcripts between 10 and 0.1 µmol O_2 L^{-1} after 60 minutes at the respective O_2 concentration. Down-regulation is depicted in blue, up-regulation in red. Asterisks depict non-significant differential expression ($P > 0.05$). 0.001 µmol O_2 L^{-1} is defined as apparent anoxia: O_2 was still supplied (10.1 µmol O_2 min^{-1}) but could no longer be accurately determined. Data for all replicates, gene locus tags and further details are listed in Table S4.

Table S1. RNA read numbers of the transcriptomes of *Acidobacterium capsulatum* 161. Triplicate total RNA samples were sequenced on an Illumina NextSeq550 High-Output sequencer (75 nucleotide read length) after rRNA depletion using the NEB Ribo-Zero rRNA removal kit for bacteria.

Strain	Samples	# of raw reads	# of reads after error correction[a]	# of reads w/o rRNA[b]	% of rRNA reads	# of reads mapped to ORFs	% of mapped reads to ORFs
Acidobacterium capsulatum 161	A-R1-10µM-t60	16281952	16222470	16016862	1.3%	11214363	70%
	A-R1-0.1µM-t60	16566631	16505408	16491155	0.1%	11415099	69.2%
	A-R1-0.001µM-t10	12566560	12528947	12499767	0.2%	8613115	68.9%
	A-R1-0.001µM-t60	13677624	13635930	13378756	1.9%	8694014	65.0%
	A-R3-10µM-t60	14757807	14683340	14634225	0.3%	9767691	66.7%
	A-R3-0.1µM-t60	13434770	13389322	13372593	0.1%	9380289	70.1%
	A-R3-0.001µM-t10	14585380	14541544	14343829	1.4%	9778275	68.2%
	A-R3-0.001µM-t60	13885090	13843635	13648069	1.4%	9088014	66.6%
	A-R4-10µM-t60	16604510	16547909	16514524	0.2%	11502196	69.6%
	A-R4-0.1µM-t60	14228960	14186186	14169379	0.1%	9862805	69.6%
	A-R4-0.001µM-t10	15232953	15184107	15167639	0.1%	10375043	68.4%
	A-R4-0.001µM-t60	15173627	15114787	14909327	1.4%	8811607	59.1%

[a]RNA reads were quality-trimmed and error corrected using BBduk and Bayes-Hammer module of SPAdes assembler. [b]rRNA reads were identified by mapping to the SILVA SSU 132, LSU 132, 5S rRNA databases. See text for details. A = one letter code for species; R = replicate; µM = µmol O_2 L^{-1}; t60/t10 = sampling timepoint after 60/10 minutes at the respective O_2 concentration.
